# Suppression of *OsMDHAR4* enhances heat tolerance by mediating H_2_O_2_-induced stomatal closure in rice plants

**DOI:** 10.1186/s12284-018-0230-5

**Published:** 2018-06-28

**Authors:** Jianping Liu, Xinjiao Sun, Feiyun Xu, Yingjiao Zhang, Qian Zhang, Rui Miao, Jianhua Zhang, Jiansheng Liang, Weifeng Xu

**Affiliations:** 1 Center for Plant Water-use and Nutrition Regulation and College of Life Sciences, Joint International Research Laboratory of Water and Nutrient in Crop, Fujian Agriculture and Forestry University, Jinshan Fuzhou, 350002 China; 2 Department of Biology, Hong Kong Baptist University, Hong Kong, China; 3 Department of Biology, Southern University of Science and Technology, Shenzhen, 518055 China

**Keywords:** Rice, Monodehydroascorbate reductase, Heat tolerance, *OsMDHAR4*, Stomata, Hydrogen peroxide

## Abstract

**Background:**

Monodehydroascorbate reductase (MDAR or MDHAR), which is responsible for growth, development and stress response in plants, is a key enzyme in the maintenance of the ascorbate acid (AsA) pool through the AsA–glutathione (AsA–GSH) cycle. High temperature affects a broad spectrum of cellular components and metabolism including AsA–GSH cycle in plants. In rice, however, the detailed roles of *OsMDHAR4* in resistance against heat stress remains unclear.

**Results:**

Here, we report that OsMDHAR4 protein was localized to the chloroplasts. *OsMDHAR4* expression was detected in all tissues surveyed and peaked in leaf blade. *OsMDHAR4* was responsive to multiple stresses and was relatively strongly induced by heat treatment. In comparison with wild type, the *osmdhar4* mutant exhibited improved tolerance to heat stress, whereas *OsMDHAR4* overexpression lines exhibited enhanced sensitivity to heat stress. Moreover, we found that suppression of *OsMDHAR4* promoted stomatal closure and hydrogen peroxide accumulation, and overexpression of *OsMDHAR4* increased stomatal opening and decreased hydrogen peroxide content in rice leaves.

**Conclusions:**

Taken together, these results indicated that *OsMDHAR4* negatively regulates tolerance to heat stress by mediating H_2_O_2_-induced stomatal closure in rice.

**Electronic supplementary material:**

The online version of this article (10.1186/s12284-018-0230-5) contains supplementary material, which is available to authorized users.

## Background

Extreme temperature is one of the serious threats affecting crop production and distribution worldwide (Yokotani et al., 2008). In plants, a transient increase of 10–15 °C above ambient, is generally considered as heat shock or heat stress, which negatively affects plant growth, seed germination, photosynthesis, respiration, water relation, and membrane stability in plants (Wahid et al., 2007), and is often accompanied by the generation of reactive oxygen species (ROS), such as hydrogen peroxide (H_2_O_2_), hydroxyl radical, superoxide anion radicals, and singlet oxygen (Liu and Huang, 2000; Mittler, 2002; Apel and Hirt, 2004; Song et al., 2014). The understanding of plant responses to heat stress in physiology, genetics, and molecular biology will be greatly helpful in improving the heat tolerance of plants through genetic engineering.

Compared with other ROS, H_2_O_2_ is more stable, more diffusive, and have a long half-life (approximately 1 ms) and high permeability across membranes (Levine et al., 1994), so it can readily escape from the organelle where it was produced to the cytosol. Previous research has shown that H_2_O_2_ plays a dual role in plants: at low concentrations, it acts as a secondary messenger involved in triggering tolerance to various biotic and abiotic stresses; but at high concentrations, it leads to programmed cell death (Quan et al., 2008). H_2_O_2_ also acts as a key regulator in a broad range of physiological processes, such as photorespiration and photosynthesis (Noctor and Foyer, 1998), growth and development (Foreman et al., 2003), the cell cycle (Mittler et al., 2004), senescence (Peng et al., 2005), and stomatal movement (Bright et al., 2006).

Stomatal pores that are located in the epidermis of plant leaves control the uptake of CO_2_ for photosynthesis and the water loss during transpiration, and play a crucial role in abiotic stress tolerance (Schroeder et al., 2001; Hetherington and Woodward, 2003). In the process of ABA-dependent stomatal closure, H_2_O_2_ plays a vital role as a signal molecule by elevating calcium levels in guard cells through the activation of plasma membrane calcium channels (Pei et al., 2000; Wang and Song, 2008). OsHTAS, a RING finger ubiquitin E3 ligase, functions in rice heat tolerance through H_2_O_2_-induced stomatal closure and is mainly involved in the ABA-dependent pathway (Liu et al., 2016). The ABA-activated SnRK2 protein kinase OPEN STOMATA1 phosphorylates NADP (NADPH) oxidase (AtrbohF), which functions to produce ABA-induced ROS in guard cells (Sirichandra et al., 2009). There’s another way of ABA-independent stomatal closure. DROUGHT AND SALT TOLERANCE (DST), a zinc finger transcription factor, negatively regulates H_2_O_2_-induced stomatal closure by directly regulating the expression of genes related to H_2_O_2_ scavenging (Huang et al., 2009). *OsSRO1c* suppresses DST to positively regulate H_2_O_2_-induced stomatal closure (You et al., 2013).

ROS is normally scavenged by an enzymatic anti-oxidative system containing catalase (CAT), ascorbate peroxidase (APX), glutathione peroxidase (GPX) and superoxide dismutase (SOD), and by a non-enzymatic anti-oxidative system including ascorbic acid (AsA), glutathione (GSH), tocopherols (TOCs) and phenolic compounds to protect plant cells. The monodehydroascorbate reductase (MDAR or MDHAR), well-known as flavin adenine dinucleotide (FAD) enzyme, is involved in the ascorbate–glutathione cycle, and plays an important role in directly reducing monodehydroascorbate (oxidized ascorbate) to ascorbate using NAD(P)H as an electron donor (Apel and Hirt 2004). MDARs were found in many eukaryotes, including cucumbers, potatoes, soybean root nodules, and rot fungus (Gill and Tuteja, 2010), and were localized in chloroplasts, mitochondria, peroxisomes, and the cytosol in plants (Omoto et al., 2013), and in microsomes, mitochondria, the Golgi apparatus, and erythrocytes in animals (Sakihama et al., 2000).

The roles of MDAR have been extensively reported under abiotic and biotic stress. Overexpression of *AtMDAR1* in tobacco confers enhanced tolerance to ozone, salt and polyethylene glycol stresses (Eltayeb et al., 2007). Overexpression of *LeMDAR* from tomato (*Lycopersicon esculentum* Mill.) enhanced tolerance to temperature and methylviologen-mediated oxidative stresses (Li et al., 2010). *OsMDHAR*-expressing yeast cells displayed enhanced tolerance to H_2_O_2_ (Kim et al., 2016). Silencing *OsMDHAR3* gene increased salt sensitivity in rice (Kim et al., 2017). Knockdown of *TaMDHAR4* and *TaMDAR6* through virus-induced gene silencing (VIGS) enhanced the wheat resistance to *Puccinia striiformis* f. sp. *tritici* (*Pst*) (Feng et al., 2014; Abou-Attia et al., 2016). However, the roles of rice MDAR in heat stress response remain unclear.

In this study, a new gene encoding the chloroplastic MDHAR (*OsMDHAR4*) from the rice plant was cloned, and its function was analyzed. The *osmdhar4* mutant displayed significantly improved heat tolerance with increased stomatal closure and reduced water loss by promoting H_2_O_2_ accumulation. *OsMDHAR4*-overexpressing transgenic plants showed obviously enhanced sensitivity to heat stress with decreased stomatal closure and accelerated water loss speed. Our results suggest that the *OsMDHAR4* gene negatively regulates resistance to heat stress in rice.

## Results

### Amino acid sequence alignment and subcellular localization of OsMDHAR4

The coding sequence (CDS) of *OsMDHAR4* gene spans 1431 bp and encodes a protein of 476 amino acids and has a predicted molecular weight of 51.86 kDa. The prediction of protein domains using NCBI and InterProScan databases indicated that OsMDHAR4 protein contains pyridine nucleotide-disulfide oxidoreductase domains (Pyr_redox_2 and NAD(P)-binding domain) and a FAD/NAD-linked reductase, dimerisation domain. The multiple amino acid sequence alignment of OsMDHAR4 and other plant MDHARs was performed using the BLAST tool at the NCBI database. OsMDHAR4 showed 87, 88, 86, 43, 69, 43, 70 and 53% identity and 92, 93, 93, 61, 80, 60, 82 and 69% similarity to ZmMDAR (*Zea mays*), BdMDAR (*Brachypodium distachyon*), TaMDHAR4, TaMDAR6 (*Triticum aestivum*), AtMDAR4, AtMDAR6 (*Arabidopsis thaliana*), GmMDAR (*Glycine max*), and SlMDAR (*Solanum lycopersicum*), respectively (Additional file 1: Figure S1).

**Fig. 1:**
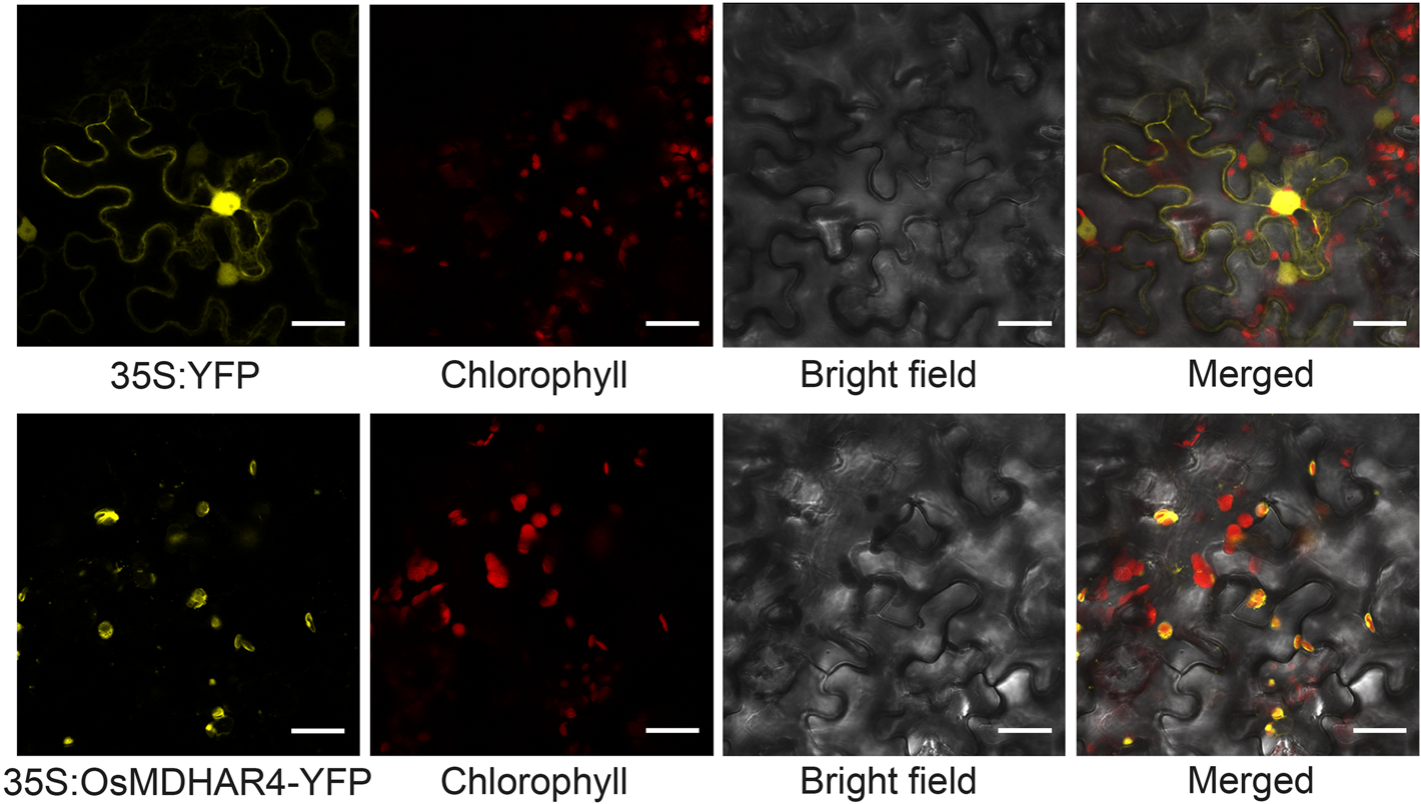
Subcellular localization of OsMDHAR4 in *Nicotiana benthamiana* by confocal fluorescent microscopy. Red color represents the autofluorescence of chlorophyll; yellow fluorescence shows the localization of OsMDHAR4-YFP. Scale bars = 20 μm

To study the subcellular localization of OsMDHAR4 protein, we fused the CDS of *OsMDHAR4* to the N-terminal of yellow fluorescent protein (YFP) driven by the cauliflower mosaic virus 35S promoter. The empty and recombinant vectors were transiently transformed into the epidermis of *Nicotiana benthamiana* by agroinfiltration. Fluorescence microscopic analysis revealed that OsMDHAR4-YFP was mainly detected in chloroplast, partially colocalized with the red autofluorescence of chlorophyll. On the contrary, 35S-YFP was widely distributed in the nucleus and cytoplasm and was not colocalized with the red autofluorescence of chloroplasts (Fig. 1). This result indicated that OsMDHAR4 protein was localized to the chloroplasts.

### Expression pattern of *OsMDHAR4*

**Fig. 2:**
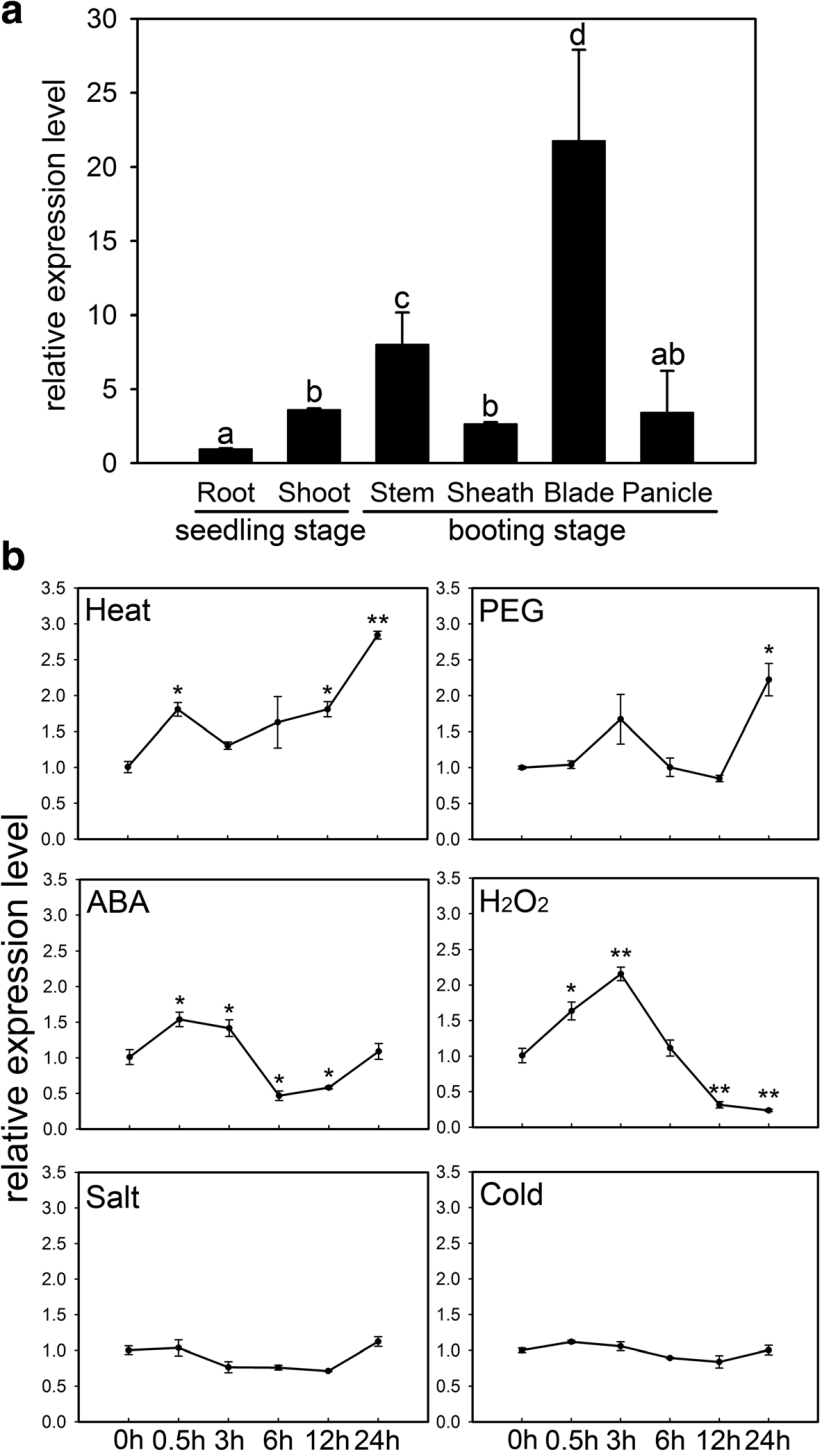
Expression pattern of *OsMDHAR4*. **a** Expression analysis of *OsMDHAR4* in different tissues containing root, shoot, stem, sheath and blade of flag leaf, and panicle by qRT-PCR. Error bars indicate the SE based on three biological replicates. Statistical differences among the samples are labeled with different letters according to the LSD test (*P* < 0.05, one-way ANOVA). **b** Expression of *OsMDHAR4* under abiotic stress conditions. Seedlings (3.5- to 4.5-leaf stage) were subjected to heat (45 °C), PEG (20% [*w*/*v*] PEG 6000), ABA (100 μM), H_2_O_2_ (100 mM), salt (250 mM NaCl), and cold (4 °C). Relative expression levels of *OsMDHAR4* were examined by qRT-PCR. The rice *Actin1* gene was used as the internal control. Error bars indicate the SE based on three biological replicates. *, *P* < 0.05, **, *P* < 0.01, by Student’s *t*-test

A total of 6 representative tissues, including root, shoot, stem, sheath and blade of flag leaf, and panicle, were prepared for spatio-temporal expression analysis of *OsMDHAR4* in rice Zhonghua11 (ZH11). The mRNA abundance of *OsMDHAR4* was detected using quantitative RT-PCR (qRT-PCR). *OsMDHAR4* transcripts were detected in all of the tissues surveyed, and the expression level was relatively lowest in root and peaked in the blade of the flag leaf (Fig. 2a).

To investigate the physiological and functional relevance of the *OsMDHAR4* gene, we further examined the expression level of *OsMDHAR4* under heat stress and other treatments, including polyethylene glycol (PEG), abscisic acid (ABA), H_2_O_2_, salt and cold, at the seedling stage. It was found that, after heat treatment, the *OsMDHAR4* transcripts increased rapidly at 0.5 h (1.8-fold), after which a slight decrease occurred at 3 h, and then they continued to rise and peaked at 24 h (2.8-fold). Under the PEG treatment, the transcript abundance of *OsMDHAR4* was nearly unchanged at most time points except for 3 h and 24 h, where two induced peaks (1.7 and 2.2-fold) appeared. In the ABA treatment, *OsMDHAR4* was induced quickly at 0.5 h and 3 h, after which a dramatic decrease appeared at 6 h and 12 h, and recovered to initial level until 24 h. When treated with H_2_O_2_, the *OsMDHAR4* expression showed a significant trend of rising followed by falling. The *OsMDHAR4* transcript level was slightly suppressed by salt and cold treatments at 6 h and 12 h (Fig. 2b). These results demonstrated that *OsMDHAR4* is responsive to multiple stresses.

### Disruption of *OsMDHAR4* increased heat tolerance

**Fig. 3:**
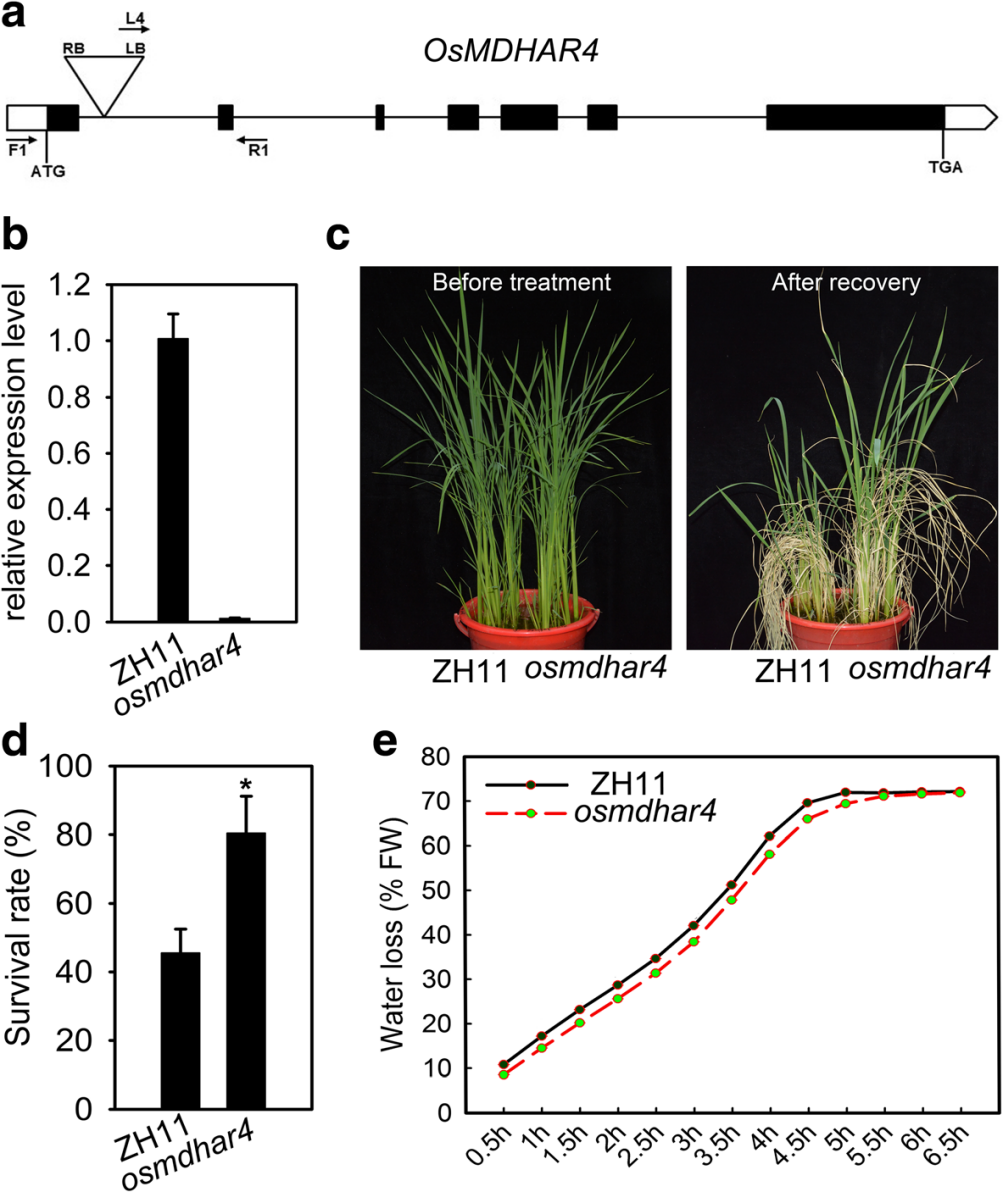
Increased heat tolerance of the *osmdhar4* mutant at seedling stage. **a** Schematic diagram of the *OsMDHAR4* gene. In the genomic DNA, exons, introns, and untranslated regions are indicated by black boxes, lines between boxes, and white boxes, respectively. The T-DNA insertion site is located in the first intron, 254 bp downstream of the start codon ATG. LB, left border of the T-DNA; RB, right border of the T-DNA. Arrows F1, R1, and L4 represent the primers used in the genotyping of the *osmdhar4* mutant. **b** Expression analysis of *OsMDHAR4* in the *osmdhar4* mutant detected by qRT-PCR. The rice *Actin1* gene was used as the internal control. Error bars indicate the SE based on three technical replicates. **c** 5.5- to 6.5-leaf stage WT and the *osmdhar4* mutant plants were growing in barrels filled with well-mixed soil. The left and right half of each barrel was planted with WT and mutant plants, respectively. Plants were treated with high temperature (45 °C) for 72 h, followed by culturing under normal conditions for 7 d. The photograph was taken before heat stress and after recovery. **d** Comparison of survival rates of WT and the *osmdhar4* mutant after heat stress. Error bars indicated the SE based on three biological replicates. *, *P* < 0.05, by Student’s *t*-test. **e** Water loss from detached leaves of WT and the *osmdhar4* mutant at indicated time points. FW, Fresh Weight

As shown in Fig. 2b, the expression of *OsMDHAR4* was significantly induced by heat treatment, and the positive roles of MDHAR have been extensively reported under abiotic stress such as ozone, salt, PEG (Sharma and Davis 1997; Eltayeb et al.,2007) and drought (Sharma and Dubey 2005). However, the role of the *MDHAR* gene in rice remains largely unknown. To evaluate the function of *OsMDHAR4* in heat stress response, we firstly searched for the Rice Mutant Database (http://rmd.ncpgr.cn/). Then, the *osmdhar4* mutant (RMD_03Z11DA36), a transfer DNA (T-DNA) insertion line in the japonica rice ZH11 background was obtained (Wu et al., 2003; Zhang et al., 2006). DNA sequencing and genotyping revealed that the T-DNA was inserted in the first intron of the *OsMDHAR4* gene, 254 bp downstream of the translation initiation site (Fig. 3a and Additional file 2: Figure S2). A qRT-PCR assay showed that *OsMDHAR4* was expressed at a very low level in the *osmdhar4* plants (Fig. 3b). Under normal growth conditions, the *osmdhar4* mutant had no obvious differences compared with the wild type (WT, ZH11) (Fig. 3c, left). *osmdhar4* and WT seedlings (5.5- to 6.5-leaf stage) were treated at high temperature (45 °C) for 72 h and subsequently returned to 26 °C for recovery. Survival rates were used as a measure of heat tolerance. After the heat treatment and recovery, 80.5% of the *osmdhar4* seedlings survived compared with 45.6% of the WT seedlings (Fig. 3c, d). Subsequently, we measured the water loss rates of detached leaves from *osmdhar4* and WT. The results showed that the detached leaves of *osmdhar4* lost water more slowly than the WT leaves (Fig. 3e). This decreased water loss of the *osmdhar4* mutant might lead to an increased tolerance to high temperature.

### Overexpression of *OsMDHAR4* decreased heat tolerance

**Fig. 4:**
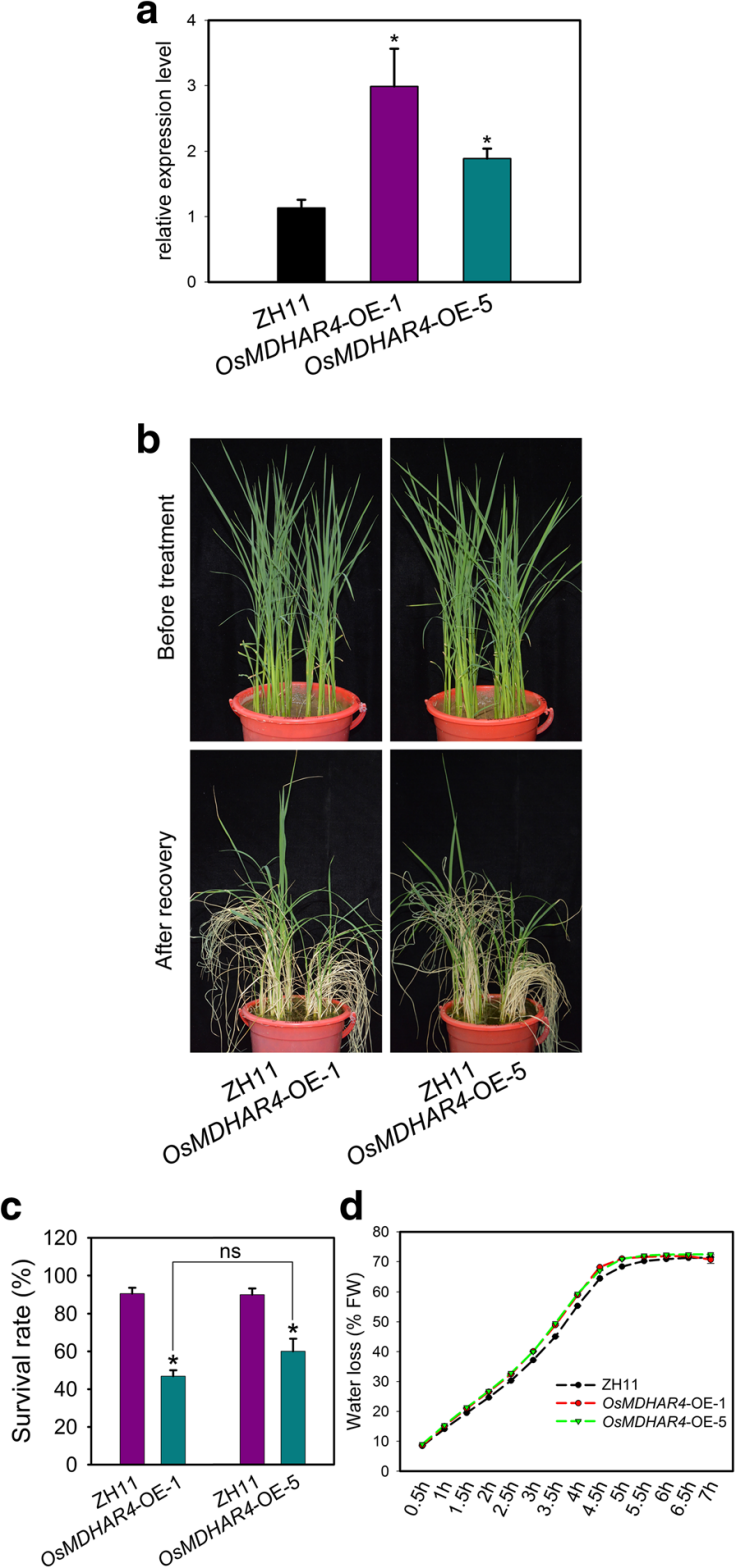
Overexpression of *OsMDHAR4* showed increased sensitivity to heat stress. **a** Transcript level of *OsMDHAR4* was measured by qRT-PCR in two *OsMDHAR4*-overexpresing lines (*OsMDHAR4*-OE-1 and *OsMDHAR4*-OE-5, ZH11 background). The rice *Actin1* gene was used as the internal control. Error bars indicate the SE based on three biological replicates.*, *P* < 0.05, by Student’s *t*-test. **b** 5.5- to 6.5-leaf stage ZH11 and *OsMDHAR4*-overexpressing plants were subjected to 45 °C high temperature environment for 48 h, followed by growing in normal conditions for 7 d. Surviving seedlings were photographed. **c** Comparison of the survival rates of ZH11 and *OsMDHAR4*-overexpressing plants after heat stress. Error bars indicated the SE based on three biological replicates. *, *P* < 0.05, by Student’s *t*-test. **d** Water loss from detached leaves of ZH11and *OsMDHAR4*-overexpressing plants at indicated time points. FW, Fresh Weight

To further validate the functions of *OsMDHAR4* in rice, we constructed an overexpression vector of *OsMDHAR4* driven by 35S promoter. The *OsMDHAR4*-overexpressing vector was transformed into rice ZH11. PCR analysis using *OsMDHAR4*-specific primers (*OsMDHAR4*-OE-F and R) and vector-specific primers (Hyg-F and R) confirmed the presence of the transgene in the two independent overexpression lines *OsMDHAR4*-OE-1 and *OsMDHAR4*-OE-5 (Additional file 3: Figure S3). Both the two transgenic lines, in which the *OsMDHAR4* expression level was significantly increased approximately triple and twice of that in WT (Fig. 4a), were selected for heat tolerance testing (45 °C for 48 h at the 5.5- to 6.5-leaf stage). Under normal conditions, we did not observe any phenotypic differences between these two overexpression lines and the WT (Fig. 4b, top). Under the heat stress treatment, both *OsMDHAR4*-OE-1 and OE-5 were more sensitive than the WT, which was consistent with the behavior of the *osmdhar4* mutant in heat treatment. The survival rates ranged from 46.9 to 60.0% for the *OsMDHAR4*-overexpressing lines, and 90.0 to 90.6% for the WT plants after recovery, but there was no significant difference between *OsMDHAR4*-OE-1 and OE-5 (Fig. 4b, c). Consistent with this result, detached leaves of *OsMDHAR4*-overexpressing lines lost water more quickly than the WT leaves (Fig. 4d). Taken together, all of these results suggested that *OsMDHAR4* plays a negative role in heat tolerance in rice.

### *OsMDHAR4* alters the stomatal aperture status of rice seedlings

**Fig. 5:**
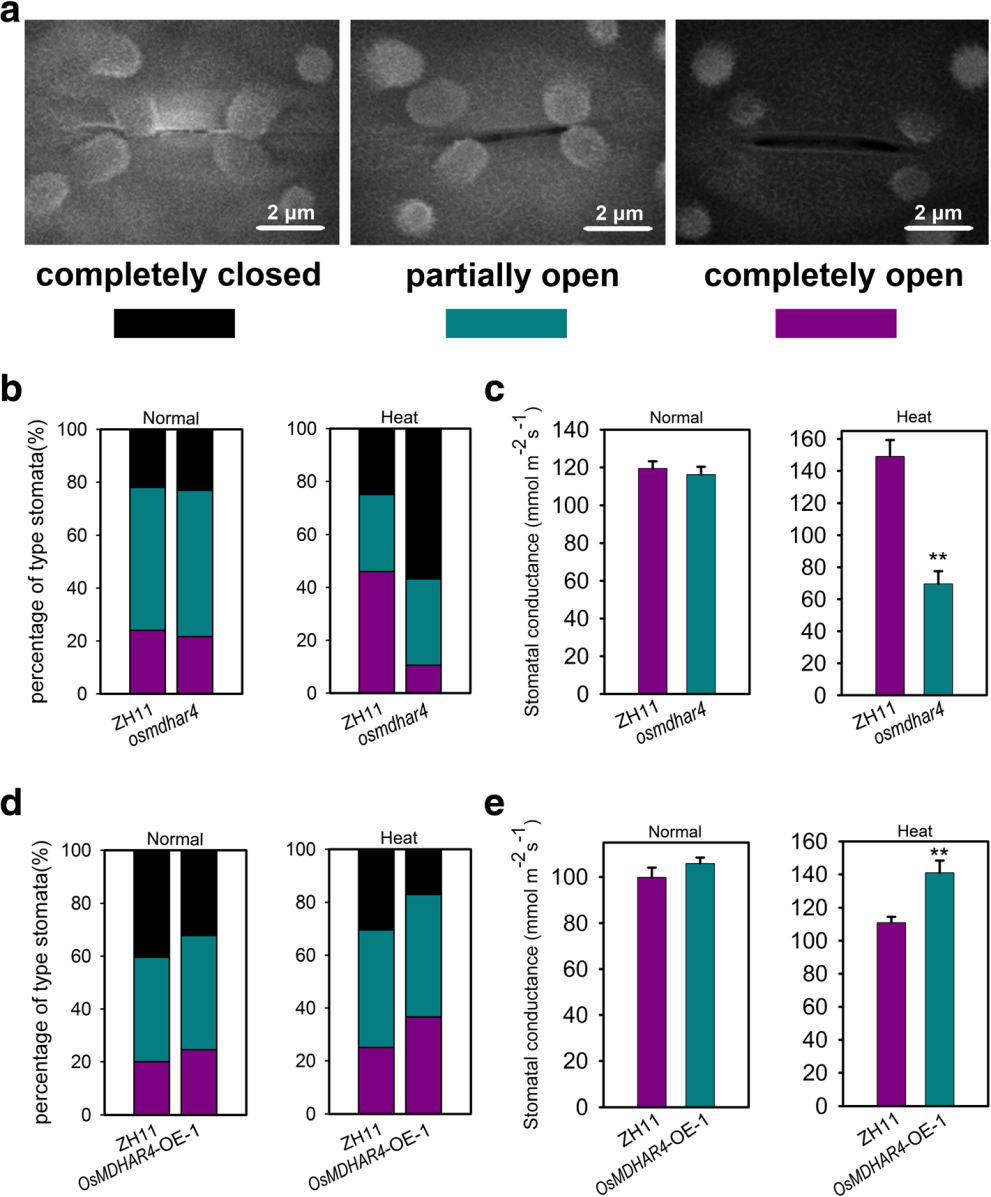
The stomatal aperture status in the *osmdhar4* mutant and *OsMDHAR4*-overexpressing plants. **a** Scanning electron microscopy images of three levels of stomatal opening. Scale bars = 2 μm. **b** Percentages of three levels of stomatal opening in WT and the *osmdhar4* mutant under normal conditions (48 stomata for WT, 40 stomata for the *osmdhar4* mutant) and heat stress conditions (28 stomata for WT, 25 stomata for the *osmdhar4* mutant). **c** Stomatal conductance of WT and *osmdhar4* mutant under normal conditions and heat stress conditions (3 repeats, 12 plants in each repeat). **d** Percentages of three levels of stomatal opening in WT and *OsMDHAR4*-OE-1 plants under normal conditions (70 stomata for WT, 90 stomata for *OsMDHAR4*-OE-1) and heat stress conditions (60 stomata for WT, 76 stomata for *OsMDHAR4*-OE-1). **e** Stomatal conductance of the WT and *OsMDHAR4*-OE-1 under normal conditions and heat stress conditions (3 repeats, 12 plants in each repeat). FW, Fresh Weight. Error bars indicate the SE based on three biological replicates. *, *P* < 0.05, and **, *P* < 0.01, by Student’s *t*-test

The function of *OsMDHAR4* in the control of water loss prompted us to investigate the stomatal aperture status, the major factor affecting the water-holding capacity in rice leaves. The stomatal apertures of the *osmdhar4* mutant, *OsMDHAR4*-OE-1 and the WT plants were checked by scanning electron microscopy. No significant differences in the percentages of the three types of stomata and stomatal conductance between the *osmdhar4* mutant and the WT were observed before heat stress. However, after heat treatment (45 °C for 24 h at 5.5- to 6.5-leaf stage), the results showed that 56.8% of stomata were completely closed in the *osmdhar4* mutant, whereas only 25.0% were completely closed in the WT plants. On the other hand, only 10.5% of stomata were completely open in the *osmdhar4* mutant, but 46% were completely open in WT. The percentage of partially open stomata didn’t differ significantly from each other (Fig. 5a, b). Furthermore, the stomatal conductance was dramatically decreased in the *osmdhar4* mutant compared the WT plants after heat treatment (Fig. 5c). These results were in agreement with the slower water loss of detached leaves from the *osmdhar4* mutant. Either before or after heat treatment, *OsMDHAR4*-OE-1 plants had lower percentages of completely closed stomata than WT (before, 32.2% for *OsMDHAR4*-OE-1, 40.4% for the WT; after, 17.2% for *OsMDHAR4*-OE-1, 30.4% for the WT), while the percentages of completely open were higher than the WT (before, 24.6% for *OsMDHAR4*-OE-1, 20.1% for the WT; after, 36.6% for *OsMDHAR4*-OE-1, 25.1% for the WT; Fig. 5d). Moreover, compared with the WT, *OsMDHAR4*-OE-1 had significantly higher stomatal conductance after heat treatment (Fig. 5e). These results were also in agreement with the faster water loss of the *OsMDHAR4*-OE-1 plants.

### *OsMDHAR4* modulates H_2_O_2_ homeostasis of rice seedlings

**Fig. 6:**
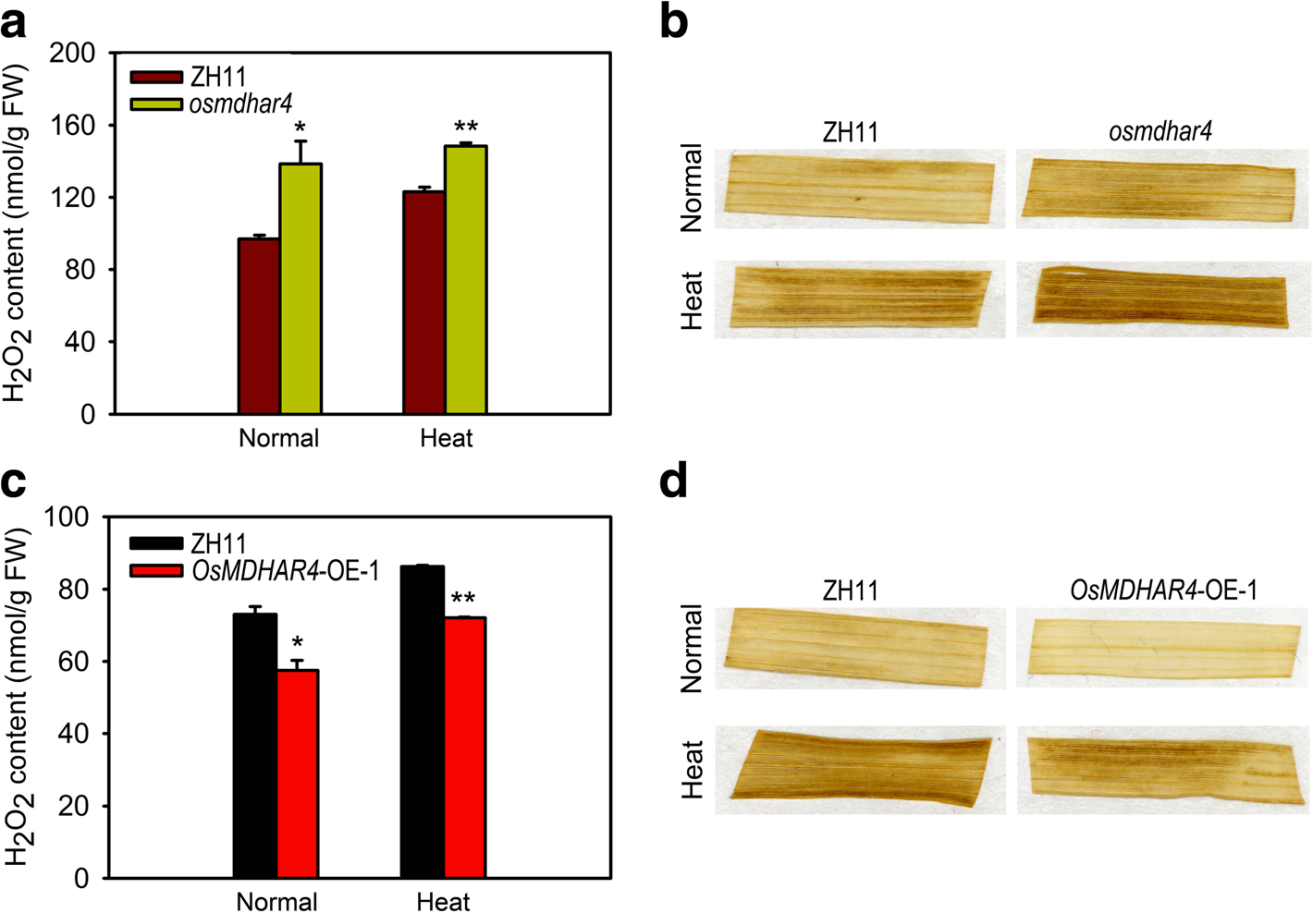
Accumulation of H_2_O_2_ in the *osmdhar4* mutant and *OsMDHAR4*-overexpressing plants. **a** Detection of the H_2_O_2_ content of WT and the *OsMDHAR4* mutant under normal and heat stress conditions. **b** DAB staining in the seedling leaves of WT and the *osmdhar4* mutant under normal and heat stress conditions. **c** Detection of the H_2_O_2_ content of WT and *OsMDHAR4*-OE-1 plants under normal and heat stress conditions. **d** DAB staining in the seedling leaves of WT and *OsMDHAR4*-OE-1 plants under normal and heat stress conditions. Error bars indicate the SE based on three biological replicates. *, *P* < 0.05, and **, *P* < 0.01, by Student’s *t*-test. FW, Fresh Weight

ROS are continuously produced as the byproducts of various metabolic pathways and are scavenged by different antioxidative defense components in plants. The equilibrium between ROS production and scavenging may be perturbed by adverse environmental factors, such as heat stress (Apel and Hirt, 2004). As H_2_O_2_ is a very stable ROS with a long half-life (Levine et al., 1994) and a signal molecular that induces stomatal closure (McAinsh et al., 1996), so we measured the H_2_O_2_ content in the rice seedling leaves. A higher level of H_2_O_2_ accumulation was detected in the *osmdhar4* mutant either before or after heat treatment (45 °C for 24 h at 5.5- to 6.5-leaf stage, Fig. 6a). The DAB staining results also support the quantitative analysis (Fig. 6b). In addition, more H_2_O_2_ accumulation was detected in the guard cells of the *osmdhar4* mutant under normal conditions, as indicated by the ROS indicator, H2DCFDA (Additional file 4: Figure S4). Thus, ROS homeostasis in *osmdhar4* seedlings was perturbed. These results indicated that the increased stomatal closure in the *osmdhar4* mutant was probably due to accumulation of H_2_O_2_ in guard cells. As expected, in the *OsMDHAR4*-OE-1 seedling leaves, visibly less H_2_O_2_ accumulation was detected under normal conditions or after heat stress through the quantitative analysis and DAB staining (Fig. 6c, d). Taken together, our results indicated that *OsMDHAR4* may negatively regulate H_2_O_2_-induced stomatal closure.

## Discussion

MDAR has been reported to be distributed widely across kingdoms pointing towards a universal role. In plants, it has been found to localize in chloroplasts, glyoxysomes, mitochondria, and cytosol. Chloroplastic MDAR localizes on the stroma of the chloroplast (Sano et al., 2005) and catalyzes the conversion of monodehydroascorbate (MDA) to AsA. AsA is a major antioxidant molecule that directly neutralizes ROS. Previous research has indicated that overexpression of chloroplastic MDAR from tomato enhanced tolerance to temperature and methyl viologen-mediated oxidative stress (Li et al., 2010). However, the physiological role of the chloroplastic MDAR in rice in response to heat stress is not reported.

In this study, *OsMDHAR4*, one new gene encoding monodehydroascorbate reductase was isolated from rice, and its amino acid sequence showed high identity and similarity to the MDAR sequences from several other plant species (Additional file 1: Figure S1). Bioinformatics studies predicted that ZmMDAR and BdMDAR, the two most homologous MDARs from *Zea mays* and *Brachypodium distachyon*, were located in the cytoplasm. However, intracellular localization studies using YFP fusion confirmed chloroplast localization of OsMDHAR4 (Fig. 1). Plant chloroplasts are the most significant generators of ROS and proposed to be heat sensors. A large number of chloroplasts are distributed in mesophyll cells (Ishikawa and Shigeoka, 2008; Maruta et al., 2012). *OsMDHAR4* transcripts peaked in the blade of flag leaf, in which a lot of mesophyll cells were distributed, while it showed the lowest transcript level in the root (almost with no chloroplasts) (Fig. 2a), the results further confirmed the finding that OsMDHAR4 protein was located in chloroplast. Since 1995, several studies have reported that MDAR genes are regulated by abiotic stresses (Leterrier et al., 2005; Grantz et al., 1995; Eltelib et al., 2011). We found that *OsMDHAR4* transcripts were induced by heat and PEG (2.8-fold and 2.2-fold). Under both ABA and H_2_O_2_ treatment, the *OsMDHAR4* expression levels were increased in first and decreased at last, but salt and cold treatments had almost no effect on these (Fig. 2b). So, we assumed that *OsMDHAR4* may function in rice heat resistance.

Many scholars have focused on conferring abiotic stress tolerance through the overexpression of *MDAR* genes. Overexpression of *LeMDAR* gene alleviated photoinhibition of PSI and PSII and enhanced the tolerance to various abiotic stresses by elevating AsA level (Li et al., 2010). Overexpressing *AeMDHAR* gene increased MDHAR enzyme activity compared to untransformed plants under both NaCl and control conditions (Sultana et al., 2012). *OsMDHAR*-expressing yeast cells displayed enhanced tolerance to H_2_O_2_ by maintaining redox homoeostasis, proteostasis, and the ascorbate (AsA)-like pool (Kim et al., 2016). In the above studies, we can find that all the *MDAR* genes play a positive role in response to different abiotic stresses. In our study, we found that disruption of *OsMDHAR4* reduced the water loss rates and increased heat tolerance (Fig. 3), and overexpression of *OsMDHAR4* accelerated the water loss rates and decreased heat tolerance (Fig. 4). Unlike previous researches, these results showed that *OsMDHAR4* plays a negative role in heat tolerance in rice.

The importance of maintaining higher levels of AsA over DHA has been reported in previous studies. In transgenic tobacco plants overexpressing cucumber ascorbate oxidase, decreased AsA/DHA ratio increased ozone sensitivity (Sanmartin et al., 2003), whereas a higher AsA/DHA brought about by overexpression of DHAR and MDAR in cytosol resulted in enhanced tolerance to salt stress (Eltayeb et al., 2006). Higher AsA/DHA ratio, higher photosynthetic activity and lower H_2_O_2_ contents were reported in transgenic tobacco plants expressing ascorbate oxidase gene (Yamamoto et al., 2005). According to this, we measured the AsA and DHA contents in leaves. Consistently with previous study (Chen et al., 2003), the *osmdhar4* mutant showed lower AsA contents and AsA/DHA ratio than the WT before or after heat treatment,and the *OsMDHAR4*-OE-1 plants showed higher AsA contents and AsA/DHA ratio than the WT before or after treatment (Additional file 5: Figure S5). However, the changes of ascorbate redox state were not be able to explain the increased heat tolerance in the *osmdhar4* mutant and incremental heat sensitivity in *OsMDHAR4* overexpression lines.

Stomata control the exchange of gases-most importantly water vapor and CO_2_-between the interior of the leaf and the atmosphere, and are involved in responses to abiotic stresses (Hetherington and Woodward, 2003). Stomatal closure degree makes major contribution to the ability of the plant to hold water in rice leaves. H_2_O_2_ is an essential signaling molecule involved in the regulation of stomatal movement (Wang and Song, 2008; Yao et al., 2013). Mutation of *DST* results in the down regulation of *peroxidase 24 precursor* (*Prx-24*, a scavenger of H_2_O_2_), might lead to the accumulation of H_2_O_2_ in guard cells and trigger stomatal closure, and enhances drought and salt tolerance (Huang et al., 2009). *Arabidopsis* mutants lacking either or both a cytosolic and chloroplastic ascorbate peroxidase (APX), which were responsible for H_2_O_2_ removal, were found to be more tolerant to salinity stress (Miller et al. 2007). Recently,one study showed that overexpression of *Prx-24* (the target gene of DST) enhanced the sensitivity to drought and salt stress in rice (Cui et al., 2015). In our case, *OsMDHAR4* might also be a target regulated by some transcript factors in response to heat stress, more research will be required to address this hypothesis. Liu et al. (2016) found that the *oshtas* mutant (gain-of-function) showed a strongly enhanced tolerance to heat stress with lower water loss rates, more closed stomata and significantly higher H_2_O_2_ contents after the treatment as compared with the WT. Our data also showed that the *osmdhar4* mutant had more closed stomata and significantly reduced stomatal conductance after heat treatment. Meanwhile, the *osmdhar4* mutant accumulated more H_2_O_2_ as compared with the WT before or after heat treatment. All of the above was found to be opposite in the *OsMDHAR4* overexpression line (*OsMDHAR4*-OE-1, Figs. 5, 6). These findings suggested that *OsMDHAR4* might play a negative regulation role in H_2_O_2_-induced stomatal closure.

## Conclusions

Our results demonstrated that *OsMDHAR4* negatively regulates tolerance to heat stress in rice by promoting H_2_O_2_-induced stomatal closure. This study increases our insights into the molecular mechanisms of rice responses to heat stress and may ultimately be helpful for enhancing heat-tolerance via genetic engineering in crop breeding programs.

## Methods

### Plant materials and stress treatments

Overexpression transgenic plants and the *osmdhar4* mutant were all based on the *Oryza sativa* L. *ssp*. Japonica Zhonghua 11 rice background.

To check the expression levels of the *OsMDHAR4* gene under various abiotic stresses or phytohormone treatment, Zhonghua 11 rice plants were grown in Yoshida solution for approximately 3 weeks under normal conditions. The seedlings at the 3.5- to 4.5-leaf stage were treated with abiotic stresses, including heat stress (exposing plants to 45 °C), simulated drought stress (treated with 20% [*w*/*v*] PEG 6000), ABA treatment (100 μM ABA), oxidative stress (treated with 100 mM H_2_O_2_), salt stress (treated with 250 mM NaCl), and cold stress (seedlings were transferred to a growth chamber at 4 °C), followed by sampling at the designated time points (0 h, 0.5 h, 3 h, 6 h, 12 h and 24 h). We prepared each RNA sample with shoots from at least four seedlings and ensured that every sampled seedling was of the same growth stage.

Before treatment, *OsMDHAR4*-overexpressing transgenic plants were firstly selected by germinated seeds on 1/2 MS medium containing 50 mg L^− 1^ hygromycin, WT and the *osmdhar4* mutant were grown on normal 1/2 MS medium 1 d later. For heat stress testing, the uniformly germinated seeds were sown in the same barrels filled with a well-mixed soil, which was then cultured in a growth chamber with a 13-h-light (28 °C)/11-h-dark (25 °C) photoperiod, 70% humidity. The 5.5- to 6.5-leaf stage seedlings were transferred to a growth chamber with a 13-h-light (45 °C)/11-h-dark (45 °C) photoperiod, 70% humidity for 48 h or 72 h. The treated seedlings were transferred back into the previous growth conditions for recovery, and seedlings with newly growing leaf blades were then counted as surviving plants.

### Plasmid construction and Rice transformation

To generate the overexpression construct, the full-length CDS of *OsMDHAR4* was amplified from the cDNA of Zhonghua 11 by PCR. The PCR fragment was inserted into vector pEXT06/g (target gene driven by 35S promoter). The resulting construct was then confirmed by sequencing and transformed into Zhonghua 11, a japonica rice that can be easily transformed, by the *Agrobacterium tumefaciens*-mediated co-cultivation method. Transformed calli were selected on hygromycin medium. The primers used in this work are listed in Additional file 6: Table S1.

### Subcellular localization of OsMDHAR4

Coding sequence of the *OsMDHAR4* gene was amplified by PCR and directionally inserted into pCAMBIA1300-35S:YFP-NOS vector. The cultures of the *Agrobacterium tumefaciens* strain EHA105 harboring empty and recombinant vector were used to infect the healthy leaves of *Nicotiana benthamiana* (4 weeks old). The fluorescence signals were observed using a confocal microscope (Leica TCS SP5 or Zeiss LSM710). The primers used in this work are listed in Additional file 6: Table S1.

### RNA extraction and qRT-PCR analysis

The TRIZol reagent (Invitrogen, USA) was used according to the manufacturer’s instructions to extract total RNA. Before reverse transcription, total RNA was treated with gDNA Eraser (TaKaRa, Japan) for 5 min at 42 °C to degrade possibly contaminated residual genomic DNA. The cDNA templates were synthesized using PrimeScript™ reagent Kit (TaKaRa, Japan) according to the manufacturer’s instructions. Quantitative real-time PCR was performed on an optical 96-well plate with a CFX96 Real-time PCR Detection System (Bio-Rad, USA) using SYBR Premix Ex Taq (TaKaRa, Japan). The PCR thermal cycling protocol was as follows: 95 °C for 10 s, followed by 40 cycles at 95 °C for 5 s and 60 °C for 30 s. Gene-specific primers for *OsMDHAR4* were designed using the Roche Web site (Roche Applied Science). The rice *Actin1* was used as the internal reference (Zhang et al., 2012), and data analyses with the 2^–ddCt^ method were performed as described (Livak and Schmittgen, 2001). The primers used in this work are listed in Additional file 6: Table S1.

### Measurement of H_2_O_2_ contents

Leaves harvested from 3.5- to 4.5-leaf stage seedlings, with or without heat treatment, were used to measure H_2_O_2_ contents. The contents were measured spectrophotometrically after reaction with potassium iodide (KI). The reaction mixture consisted of 0.5 ml of 0.1% trichloroacetic acid (TCA), leaf extract supernatant, 0.5 ml of 100 mM potassium phosphate buffer (pH 7.8) and 1 ml reagent (1 M KI, *w*/*v* in fresh double-distilled water). The blank control consisted of 1 ml 0.1% TCA and 1 ml KI in the absence of leaf extract. After 1 h of reaction in darkness, the absorbance was measured at 390 nm. The amount of H_2_O_2_ was calculated using a standard curve prepared with known concentrations of H_2_O_2_.

### DAB staining for H_2_O_2_ in leaves and detection of H_2_O_2_ production in guard cells

3,3′-Diaminobenzidine (DAB) staining was performed following a published method with some modifications (Liu et al., 2014). Rice seedlings at 5.5- to 6.5-leaf stage were treated with a 45 °C high temperature for 24 h or not treated, then the leaves (3–5 mm in width) at the same position were detached and immersed in 1% 3,3′-diaminobenzidine (DAB) solution in HCl-acidifed (pH 3.8). After 30 min under vacuum the samples were incubated at room temperature for 24 h in the dark. The samples were then bleached by boiling in ethanol in order to remove the chlorophyll and reveal the brown spots, which are indicative of the reaction of DAB with H_2_O_2_. The samples were observed and imaged under a scanner.

H_2_O_2_ production in guard cells was detected using 2′,7′-dichlorodihydrofluorescein diacetate (H2DCFDA; Molecular Probes) as described previously (Huang et al., 2009) with small modifications. Leaves of 5.5- to 6.5-leaf stage seedlings were immerged in 0.01% Tween-20 and vacuum-infiltrated for 10 min. After rinsing twice with distilled water, leaves were incubated in 3% (*w*/*v*) cellulase RS (Yakult Honsha, Japan) for 5 h at 40 °C without shaking to facilitate peeling off the epidermal strips. The epidermal strips were peeled off from leaves using tissue forceps. After washing with loading buffer (10 mM Tris-HCl, 50 mM KCl at pH 7.2), the epidermal strips were incubated in staining buffer (loading buffer containing 50 mM H2DCFDA) for 10 min at room temperature in the dark. The epidermal strips were washed with distilled water to remove the excess dye. The fluorescence was examined using a confocal laser-scanning microscope (Leica TCS SP5, Germany). All confocal images were taken under identical conditions and the guard cell region was selected to quantify the mean grey value of guard cells.

### Observation of guard cells and detection of water loss rates

Leaves of 3.5- to 4.5-leaf stage plants with or without heat treatment for 24 h were fixed with 2.5% (*v*/v) glutaraldehyde, and stomatal images (20 kV, 2000**×**) were obtained using scanning electron microscopy (KYKY-EM3200).

Plants germinated under normal conditions for 4 weeks. The leaves were detached from various lines with same age and position, and weighed immediately as the initial fresh weight. They were then placed in clean filter papers, and incubated at 25 °C. The decreases in fresh weight were recorded at every 30 min for 6.5 h or 7 h. Water loss was presented as percentage of fresh weight loss versus the initial fresh weight.

### Measurement of the AsA and DHA contents

Leaves harvested from 3.5- to 4.5-leaf stage seedlings, with or without heat treatment, were used to measure the total AsA (tAsA), AsA and DHA contents determined by the spectrophotometric method described previously (Gillespie and Ainsworth, 2007), with minor modifications. The frozen leaf samples were ground with inert sand and 10% trichloroacetic acid (TCA) solution using a mortar and pestle. The homogenate was centrifuged at 12,000 rpm for 20 min. The tAsA contents were determined in a reaction mixture consisting of crude extract and 150 mM KH_2_PO_4_ buffer (pH 7.4) containing 5 mM EDTA and 10 mM dithiothreitol (DTT) for the reduction of DHA to AsA. The reaction mixtures were incubated at room temperature for 10 min and 0.5% N-ethylmaleimide (NEM) was added. AsA was assayed in a similar manner, except that deionized H_2_O was substituted for DTT and NEM. The color was developed in both the reaction mixtures by the addition of 10% TCA, 44% *o*-phosphoric acid, α,ά-dipyridyl in 70% ethanol, and 30% FeCl_3_. The reaction mixtures were incubated at 37 °C for 1 h and quantified spectrophotometrically at 525 nm.

## Additional files

Additional file 1: **Figure S1.** Alignment of OsMDHAR4 protein with monodehydroascorbate reductase proteins from *Zea mays* (ZmMDAR), *Brachypodium distachyon* (BdMDAR), *Triticum aestivum* (TaMDHAR4, TaMDAR6), *Arabidopsis thaliana* (AtMDAR4, AtMDAR6), *Glycine max* (GmMDAR), and *Solanum lycopersicum* (SlMDAR). Red line: FAD/NAD-binding domain, blue line: Pyr_redox_2 (pyridine nucleotide-disulfide oxidoreductase 2) domain, and green line: FAD/NAD-linked reductase, domain. Dark blue shadow: amino acids are conserved among all 9 sequences, pink shadow: amino acids are conserved among 7 or 8 sequences, light blue shadow: amino acids are conserved among 5 or 6 sequences Sequence alignment was performed using the DNAMAN6 software. (JPG 9854 kb)

Additional file 2: **Figure S2.** Genotyping of *osmdhar4* T-DNA insertion mutant. F1, R1 and L4 stand for the primers. W, wild type. M, homozygous mutant. H, heterozygous mutant. (JPG 479 kb)

Additional file 3: **Figure S3. a** *OsMDHAR4*-overexpressing construct. **b** PCR identification of transgenic plants from two overexpression lines (*OsMDHAR4*-OE-1 and OE-5). Positive transgenic plants have a PCR band about 1471 bp or 675 bp when using different primers combinations (*OsMDHAR4*-OE-F + R or Hyg-F + R). P, positive control. N, negative control. (JPG 420 kb)

Additional file 4: **Figure S4. a** Detection of H_2_O_2_ production in guard cells of WT and *osmdhar4* mutant plants with H2DCFDA. Scale bars = 10 μm. **b** Quantitative analysis of H_2_O_2_ production in guard cells of WT and *osmdhar4* mutant (3 repeats, 12 stomata in each repeat). Error bars indicate the SE based on three biological replicates. *, *P* < 0.05, by Student’s *t*-test. (JPG 2714 kb)

Additional file 5: **Figure S5. a** Detection of the AsA content of the WT, the *osmdhar4* mutant and *OsMDHAR4*-OE-1 plants under normal or heat stress conditions. **b** Detection of the DHA content of the WT, the *osmdhar4* mutant and *OsMDHAR4*-OE-1 plants under normal or heat stress conditions. **c** AsA/DHA ratio of WT, the *osmdhar4* mutant and *OsMDHAR4*-OE-1 plants under normal or heat stress conditions. Error bars indicate the SE based on three biological replicates. *, *P* < 0.05, **, *P* < 0.01, by Student’s *t*-test. ns, no significant. FW, Fresh Weight. (JPG 480 kb)

Additional file 6: **Table S1** List of primers used in this study (F, forward primer; R, reverse primer; q, quantitative RT-PCR). (DOCX 23 kb)

## Abbreviations

ABA: Abscisic acid
APX: Ascorbate peroxidase
AsA: Ascorbic acid
CAT: Catalase
CDS: Coding sequence
DAB: 3,3′-Diaminobenzidine
DHA: Dehydroascorbate
FAD: Flavin adeninedinucleotide
GPX: Glutathione peroxidase
GSH: Glutathione
H2DCFDA: 2′,7′-dichlorodihydrofluorescein diacetate
H_2_O_2_: Hydrogen peroxide
Hyg: Hygromycin
MDA: Monodehydroascorbate
MDAR or MDHAR: Monodehydroascorbate reductase
OE: Overexpression
PEG: Polyethylene glycol
qRT-PCR: Quantitative real time polymerase chain reaction
ROS: Reactive oxygen species
SOD: Superoxide dismutase
TOCs: Tocopherols
YFP: Yellow fluorescent protein
ZH11: Zhonghua 11

## References

[CR1] Abou-Attia MA, Wang X, Nashaat Al-Attala M, Xu Q, Zhan G, Kang Z (2016). *TaMDAR6* acts as a negative regulator of plant cell death and participates indirectly in stomatal regulation during the wheat stripe rust-fungus interaction. Physiol Plant.

[CR2] Apel K, Hirt H (2004). Reactive oxygen species: metabolism, oxidative stress, and signal transduction. Annu Rev Plant Biol.

[CR3] Bright J, Desikan R, Hancock JT, Weir IS, Neill SJ (2006). ABA-induced NO generation and stomatal closure in *Arabidopsis* are dependent on H_2_O_2_ synthesis. Plant J.

[CR4] Chen Z, Young TE, Ling J, Chang SC, Gallie DR (2003). Increasing vitamin C content of plants through enhanced ascorbate recycling. Proc Natl Acad Sci.

[CR5] Cui LG, Shan JX, Shi M, Gao JP, Lin HX (2015). DCA1 acts as a transcriptional co-activator of DST and contributes to drought and salt tolerance in Rice. PLoS Genet.

[CR6] Eltayeb AE, Kawano N, Badawi GH, Kaminaka H, Sanekata T, Morishima I, Shibahara T, Inanaga S, Tanaka K (2006). Enhanced tolerance to ozone and drought stresses in transgenic tobacco overexpressing dehydroascorbate reductase in cytosol. Physiol Plant.

[CR7] Eltayeb AE, Kawano N, Badawi GH, Kaminaka H, Sanekata T, Shibahara T, Inanaga S, Tanaka K (2007). Overexpression of monodehydroascorbate reductase in transgenic tobacco confers enhanced tolerance to ozone. salt and polyethylene glycol stresses Planta.

[CR8] Eltelib HA, Badejo AA, Fujikawa Y, Esaka M (2011). Gene expression of monodehydroascorbate reductase and dehydroascorbate reductase during fruit ripening and in response to environmental stresses in acerola (*Malpighia glabra*). J Plant Physiol.

[CR9] Feng H, Liu W, Zhang Q, Wang X, Wang X, Duan X, Li F, Huang L, Kang Z (2014). *TaMDHAR4*, a monodehydroascorbate reductase gene participates in the interactions between wheat and *Puccinia striiformis* f. Sp. *tritici*. Plant Physiol Biochem.

[CR10] Foreman J, Demidchik V, Bothwell JH, Mylona P, Miedema H, Torres MA, Linstead P, Costa S, Brownlee C, Jones JD, Davies JM, Dolan L (2003). Reactive oxygen species produced by NADPH oxidase regulate plant cell growth. Nature.

[CR11] Gill SS, Tuteja N (2010). Reactive oxygen species and antioxidant machinery in abiotic stress tolerance in crop plants. Plant Physiol Biochem.

[CR12] Gillespie KM, Ainsworth EA (2007). Measurement of reduced, oxidized and total ascorbate content in plants. Nat Protoc.

[CR13] Grantz AA, Brummell DA, Bennett AB (1995). Ascorbate free radical reductase mRNA levels are induced by wounding. Plant Physiol.

[CR14] Hetherington AM, Woodward FI (2003). The role of stomata in sensing and driving environmental change. Nature.

[CR15] Huang XY, Chao DY, Gao JP, Zhu MZ, Shi M, Lin HX (2009). A previously unknown zinc finger protein, DST, regulates drought and salt tolerance in rice via stomatal aperture control. Genes Dev.

[CR16] Ishikawa T, Shigeoka S (2008). Recent advances in ascorbate biosynthesis and the physiological significance of ascorbate peroxidase in photosynthesizing organisms. Biosci Biotechnol Biochem.

[CR17] Kim IS, Kim YS, Kim YH, Park AK, Kim HW, Lee JH, Yoon HS (2016). Potential application of the Oryza sativa Monodehydroascorbate reductase gene (*OsMDHAR*) to improve the stress tolerance and fermentative capacity of Saccharomyces cerevisiae. PLoS One.

[CR18] Kim JJ, Kim YS, Park SI, Mok JE, Kim YH, Park HM, Kim IS, Yoon HS (2017) Cytosolic monodehydroascorbate reductase gene affects stress adaptation and grain yield under paddy field conditions in *Oryza sativa* L*. japonica*. Mol Breed 37

[CR19] Leterrier M, Corpas FJ, Barroso JB, Sandalio LM, del Rio LA (2005). Peroxisomal monodehydroascorbate reductase. Genomic clone characterization and functional analysis under environmental stress conditions. Plant Physiol.

[CR20] Levine A, Tenhaken R, Dixon R, Lamb C (1994). H_2_O_2_ from the oxidative burst orchestrates the plant hypersensitive disease resistance response. Cell.

[CR21] Li F, Wu QY, Sun YL, Wang LY, Yang XH, Meng QW (2010). Overexpression of chloroplastic monodehydroascorbate reductase enhanced tolerance to temperature and methyl viologen-mediated oxidative stresses. Physiol Plant.

[CR22] Liu H, Ma Y, Chen N, Guo S, Liu H, Guo X, Chong K, Xu Y (2014). Overexpression of stress-inducible OsBURP16, the beta subunit of polygalacturonase 1, decreases pectin content and cell adhesion and increases abiotic stress sensitivity in rice. Plant Cell Environ.

[CR23] Liu J, Zhang C, Wei C, Liu X, Wang M, Yu F, Xie Q, Tu J (2016). The RING finger ubiquitin E3 ligase OsHTAS enhances heat tolerance by promoting H_2_O_2_-induced stomatal closure in Rice. Plant Physiol.

[CR24] Liu X, Huang B (2000). Heat stress injury in relation to membrane lipid peroxidation in creeping Bentgrass. Crop Sci.

[CR25] Livak KJ, Schmittgen TD (2001). Analysis of relative gene expression data using real-time quantitative PCR and the 2^-ΔΔCT^ method. Methods.

[CR26] Maruta T, Noshi M, Tanouchi A, Tamoi M, Yabuta Y, Yoshimura K, Ishikawa T, Shigeoka S (2012). H_2_O_2_-triggered retrograde signaling from chloroplasts to nucleus plays specific role in response to stress. J Biol Chem.

[CR27] McAinsh MR, Clayton H, Mansfield TA, Hetherington AM (1996). Changes in stomatal behavior and guard cell cytosolic free calcium in response to oxidative stress. Plant Physiol.

[CR28] Miller G, Suzuki N, Rizhsky L, Hegie A, Koussevitzky S, Mittler R (2007). Double mutants deficient in cytosolic and thylakoid ascorbate peroxidase reveal a complex mode of interaction between reactive oxygen species, plant development. and response to abiotic stresses Plant Physiol.

[CR29] Mittler R (2002). Oxidative stress. antioxidants and stress tolerance Trends Plant Sci.

[CR30] Mittler R, Vanderauwera S, Gollery M, Van Breusegem F (2004). Reactive oxygen gene network of plants. Trends Plant Sci.

[CR31] Noctor G, Foyer CH (1998). ASCORBATE AND GLUTATHIONE: keeping active oxygen under control. Annu Rev Plant Physiol Plant Mol Biol.

[CR32] Omoto E, Nagao H, Taniguchi M, Miyake H (2013). Localization of reactive oxygen species and change of antioxidant capacities in mesophyll and bundle sheath chloroplasts of maize under salinity. Physiol Plant.

[CR33] Pei ZM, Murata Y, Benning G, Thomine S, Klusener B, Allen GJ, Grill E, Schroeder JI (2000). Calcium channels activated by hydrogen peroxide mediate abscisic acid signalling in guard cells. Nature.

[CR34] Peng C, Ou Z, Liu N, Lin G (2005). Response to high temperature in flag leaves of super high-yielding rice Pei’ai 64S/E32 and Liangyoupeijiu. Rice Sci.

[CR35] Quan LJ, Zhang B, Shi WW, Li HY (2008). Hydrogen peroxide in plants: a versatile molecule of the reactive oxygen species network. J Integr Plant Biol.

[CR36] Sakihama Y, Mano J, Sano S, Asada K, Yamasaki H (2000). Reduction of phenoxyl radicals mediated by monodehydroascorbate reductase. Biochem Biophys Res Commun.

[CR37] Sanmartin M, Drogoudi PD, Lyons T, Pateraki I, Barnes J, Kanellis AK (2003). Over-expression of ascorbate oxidase in the apoplast of transgenic tobacco results in altered ascorbate and glutathione redox states and increased sensitivity to ozone. Planta.

[CR38] Sano S, Tao S, Endo Y, Inaba T, Hossain MA, Miyake C, Matsuo M, Aoki H, Asada K, Saito K (2005). Purification and cDNA cloning of chloroplastic monodehydroascorbate reductase from spinach. Biosci Biotechnol Biochem.

[CR39] Schroeder JI, Allen GJ, Hugouvieux V, Kwak JM, Waner D (2001). Guard cell signal transduction. Annu Rev Plant Biol.

[CR40] Sharma P, Dubey RS (2005). Modulation of nitrate reductase activity in rice seedlings under aluminium toxicity and water stress: role of osmolytes as enzyme protectant. J Plant Physiol.

[CR41] Sharma YK, Davis KR (1997). The effects of ozone on antioxidant responses in plants. Free Radic Biol Med.

[CR42] Sirichandra C, Gu D, Hu H-C, Davanture M, Lee S, Djaoui M, Valot B, Zivy M, Leung J, Merlot S (2009). Phosphorylation of the *Arabidopsis* AtrbohF NADPH oxidase by OST1 protein kinase. FEBS Lett.

[CR43] Song Y, Miao Y, Song CP (2014). Behind the scenes: the roles of reactive oxygen species in guard cells. New Phytol.

[CR44] Sultana S, Khew CY, Morshed MM, Namasivayam P, Napis S, Ho CL (2012). Overexpression of monodehydroascorbate reductase from a mangrove plant (AeMDHAR) confers salt tolerance on rice. J Plant Physiol.

[CR45] Wahid A, Gelani S, Ashraf M, Foolad MR (2007). Heat tolerance in plants: an overview. Environ Exp Bot.

[CR46] Wang P, Song CP (2008). Guard-cell signalling for hydrogen peroxide and abscisic acid. New Phytol.

[CR47] Wu C, Li X, Yuan W, Chen G, Kilian A, Li J, Xu C, Li X, Zhou DX, Wang S, Zhang Q (2003). Development of enhancer trap lines for functional analysis of the rice genome. Plant J.

[CR48] Yamamoto A, Bhuiyan MN, Waditee R, Tanaka Y, Esaka M, Oba K, Jagendorf AT, Takabe T (2005). Suppressed expression of the apoplastic ascorbate oxidase gene increases salt tolerance in tobacco and Arabidopsis plants. J Exp Bot.

[CR49] Yao Y, Liu X, Li Z, Ma X, Rennenberg H, Wang X, Li H (2013). Drought-induced H_2_O_2_ accumulation in subsidiary cells is involved in regulatory signaling of stomatal closure in maize leaves. Planta.

[CR50] Yokotani N, Ichikawa T, Kondou Y, Matsui M, Hirochika H, Iwabuchi M, Oda K (2008). Expression of rice heat stress transcription factor OsHsfA2e enhances tolerance to environmental stresses in transgenic *Arabidopsis*. Planta.

[CR51] You J, Zong W, Li X, Ning J, Hu H, Li X, Xiao J, Xiong L (2013). The SNAC1-targeted gene *OsSRO1c* modulates stomatal closure and oxidative stress tolerance by regulating hydrogen peroxide in rice. J Exp Bot.

[CR52] Zhang CC, Yuan WY, Zhang QF (2012). *RPL1*, a gene involved in epigenetic processes regulates phenotypic plasticity in rice. Mol Plant.

[CR53] Zhang J, Li C, Wu C, Xiong L, Chen G, Zhang Q, Wang S (2006). RMD: a rice mutant database for functional analysis of the rice genome. Nucleic Acids Res.

