# Supplementary figures and images for: Suppression of OsMDHAR4 enhances heat tolerance by mediating H2O2-induced stomatal closure in rice plants
Source: Rice (N Y). 2018 Jun 28;11:38. doi: 10.1186/s12284-018-0230-5 (PMC6021276; doi:10.1186/s12284-018-0230-5)

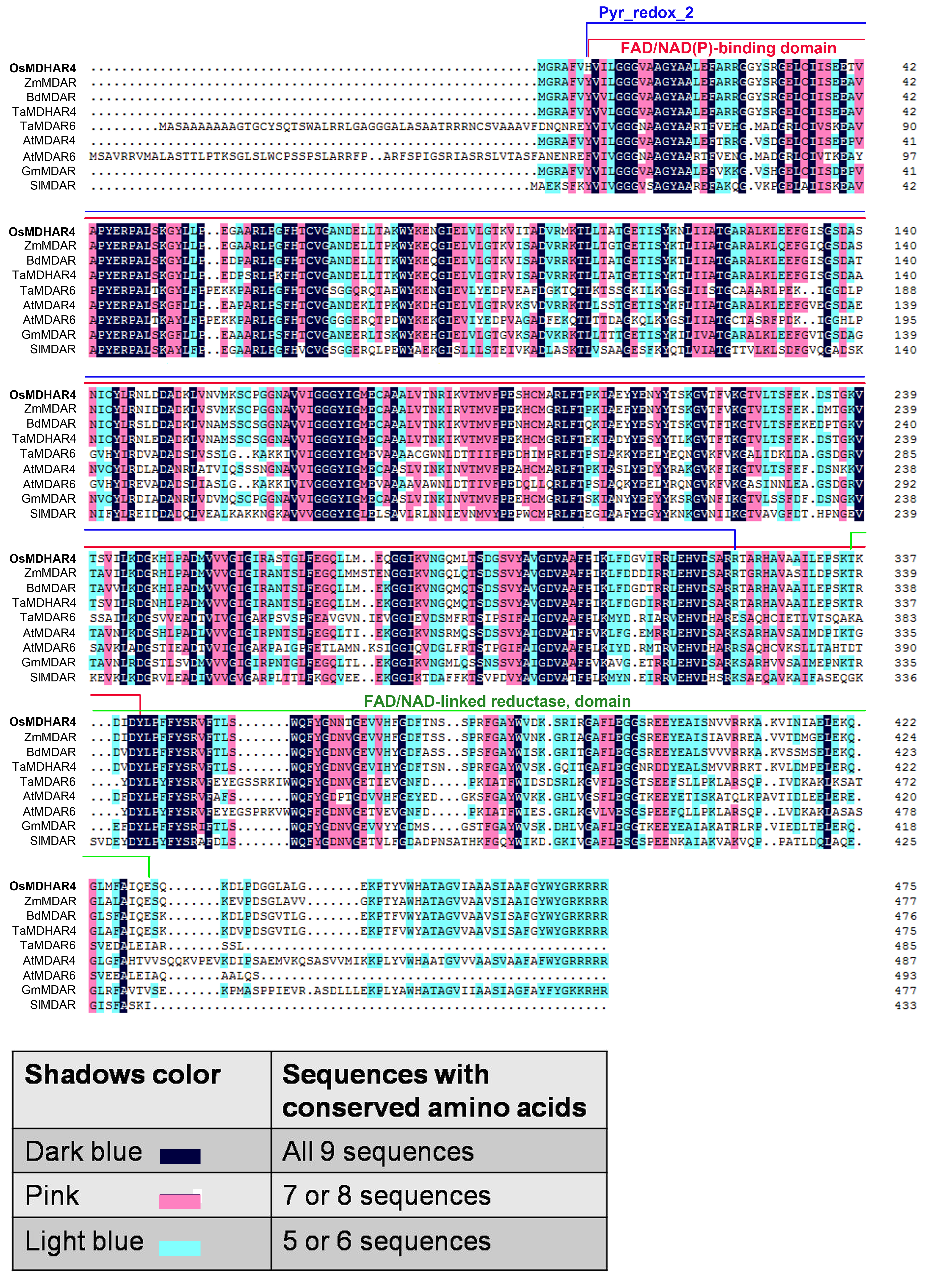

Supplement: Supplementary file 1 — Figure S1. Alignment of OsMDHAR4 protein with monodehydroascorbate reductase proteins from Zea mays (ZmMDAR), Brachypodium distachyon (BdMDAR), Triticum aestivum (TaMDHAR4, TaMDAR6), Arabidopsis thaliana (AtMDAR4, AtMDAR6), Glycine max (GmMDAR), and Solanum lycopersicum (SlMDAR). Red line: FAD/NAD-binding domain, blue line: Pyr_redox_2 (pyridine nucleotide-disulfide oxidoreductase 2) domain, and green line: FAD/NAD-linked reductase, domain. Dark blue shadow: amino acids are conserved among all 9 sequences, pink shadow: amino acids are conserved among 7 or 8 sequences, light blue shadow: amino acids are conserved among 5 or 6 sequences Sequence alignment was performed using the DNAMAN6 software. (JPG 9854 kb) [file 12284_2018_230_MOESM1_ESM.jpg]

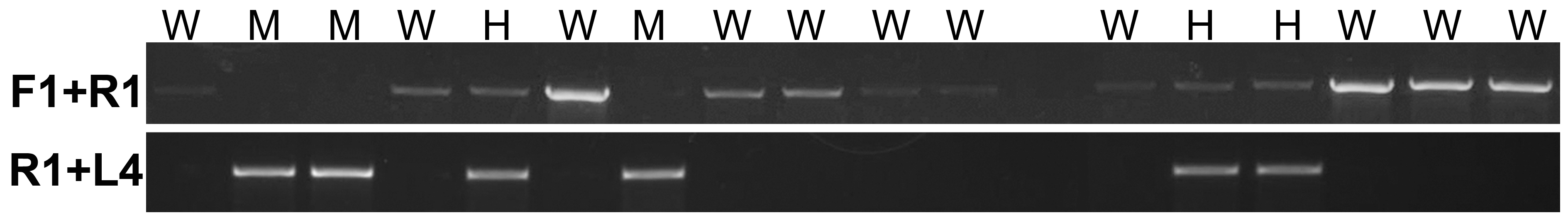

Supplement: Supplementary file 2 — Figure S2. Genotyping of osmdhar4 T-DNA insertion mutant. F1, R1 and L4 stand for the primers. W, wild type. M, homozygous mutant. H, heterozygous mutant. (JPG 479 kb) [file 12284_2018_230_MOESM2_ESM.jpg]

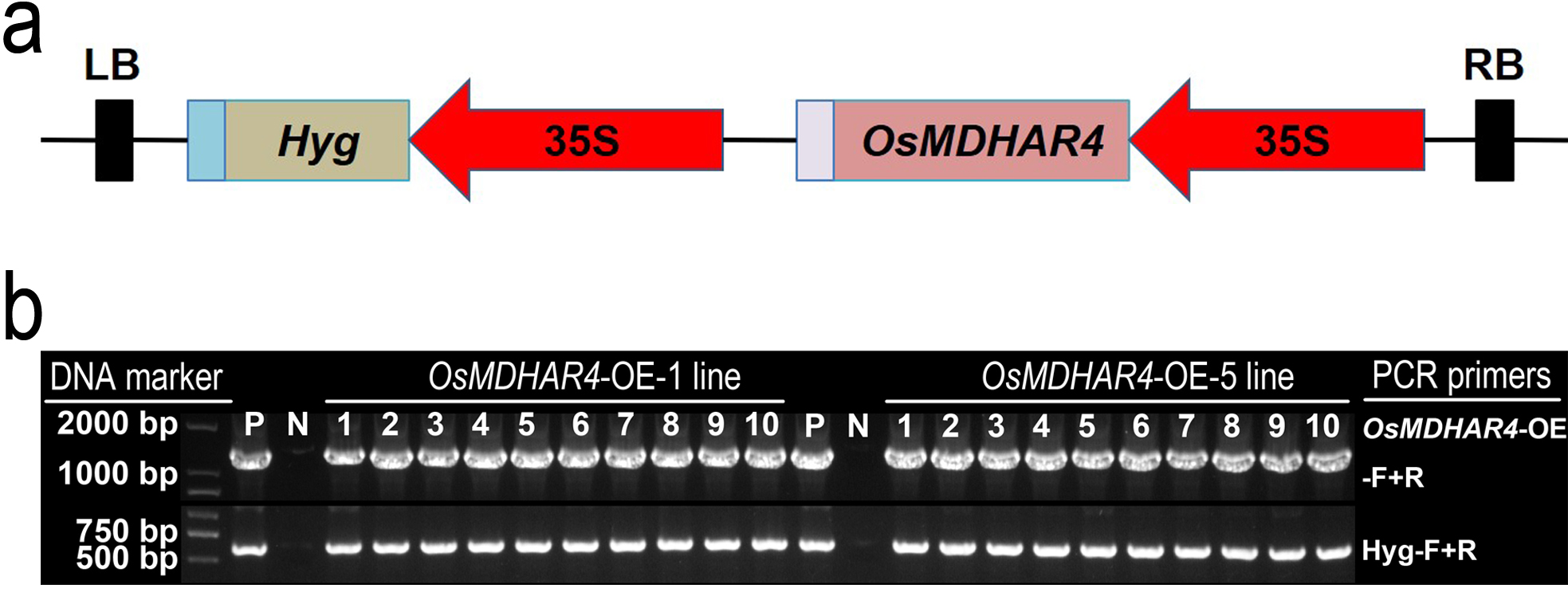

Supplement: Supplementary file 3 — Figure S3. a OsMDHAR4-overexpressing construct. b PCR identification of transgenic plants from two overexpression lines (OsMDHAR4-OE-1 and OE-5). Positive transgenic plants have a PCR band about 1471 bp or 675 bp when using different primers combinations (OsMDHAR4-OE-F + R or Hyg-F + R). P, positive control. N, negative control. (JPG 420 kb) [file 12284_2018_230_MOESM3_ESM.jpg]

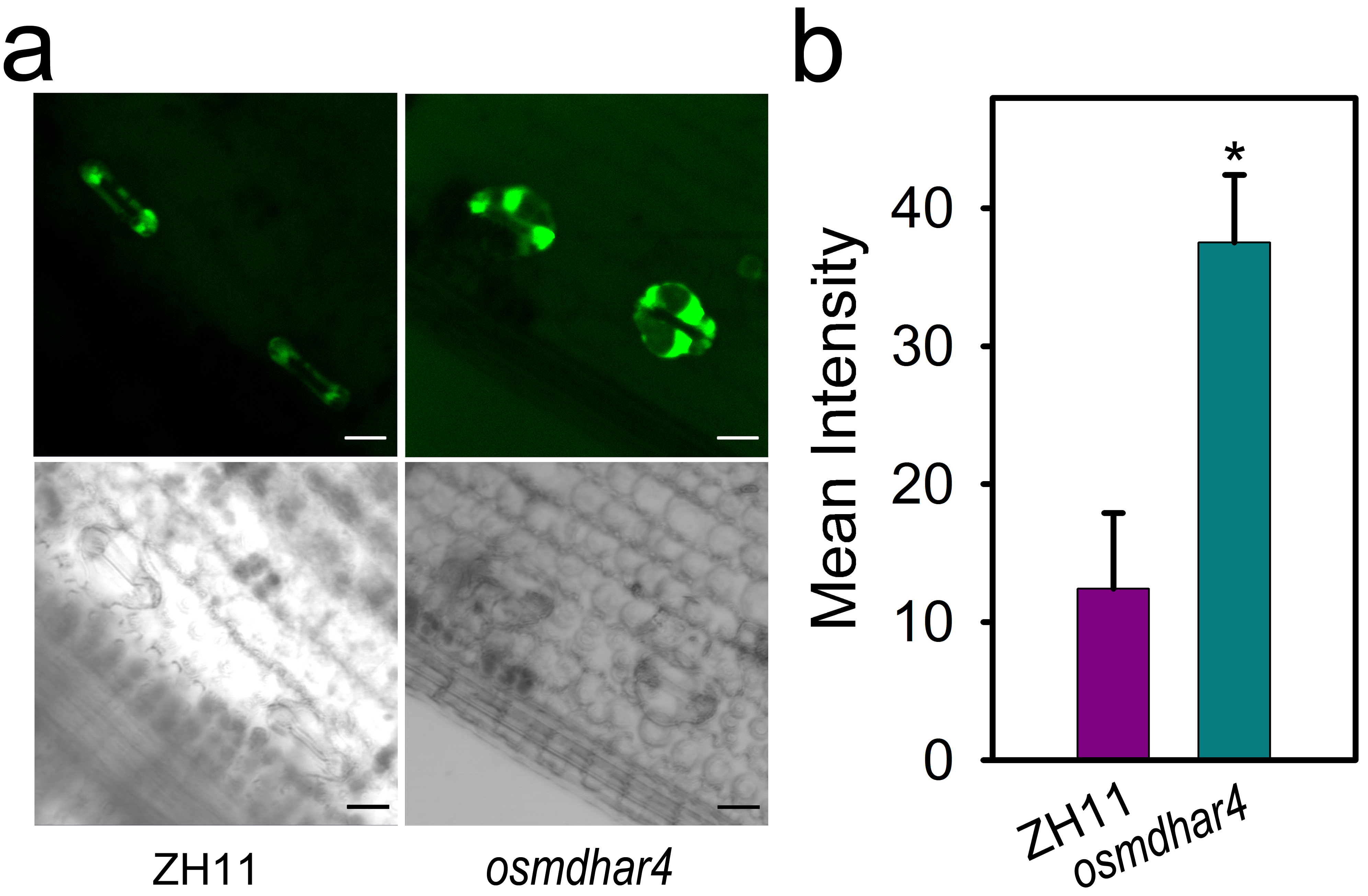

Supplement: Supplementary file 4 — Figure S4. a Detection of H2O2 production in guard cells of WT and osmdhar4 mutant plants with H2DCFDA. Scale bars = 10 μm. b Quantitative analysis of H2O2 production in guard cells of WT and osmdhar4 mutant (3 repeats, 12 stomata in each repeat). Error bars indicate the SE based on three biological replicates. *, P < 0.05, by Student’s t-test. (JPG 2714 kb) [file 12284_2018_230_MOESM4_ESM.jpg]

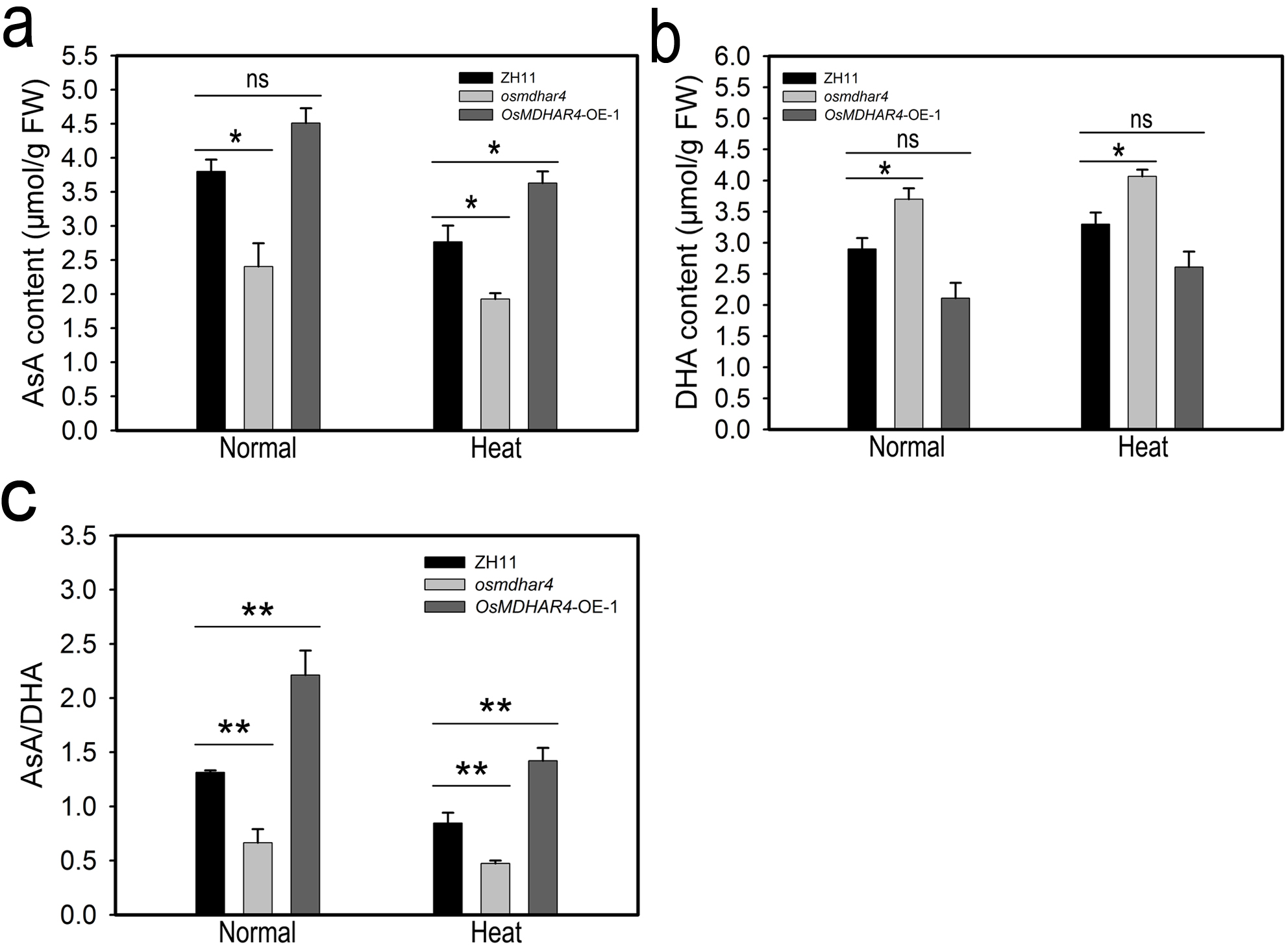

Supplement: Supplementary file 5 — Figure S5. a Detection of the AsA content of the WT, the osmdhar4 mutant and OsMDHAR4-OE-1 plants under normal or heat stress conditions. b Detection of the DHA content of the WT, the osmdhar4 mutant and OsMDHAR4-OE-1 plants under normal or heat stress conditions. c AsA/DHA ratio of WT, the osmdhar4 mutant and OsMDHAR4-OE-1 plants under normal or heat stress conditions. Error bars indicate the SE based on three biological replicates. *, P < 0.05, **, P < 0.01, by Student’s t-test. ns, no significant. FW, Fresh Weight. (JPG 480 kb) [file 12284_2018_230_MOESM5_ESM.jpg]
