# Supplementary material for: Suppression of OsMDHAR4 enhances heat tolerance by mediating H2O2-induced stomatal closure in rice plants
Source: Rice (N Y). 2018 Jun 28;11:38. doi: 10.1186/s12284-018-0230-5 (PMC6021276; doi:10.1186/s12284-018-0230-5)
Supplement: Supplementary file 6 — Table S1 List of primers used in this study (F, forward primer; R, reverse primer; q, quantitative RT-PCR). (DOCX 23 kb) [file 12284_2018_230_MOESM6_ESM.docx]

**Table S1** List of primers used in this study (F, forward primer; R, reverse primer; q, quantitative real-time PCR)

| **Primer name** | **Primer sequence (5′→ 3′)** |
| --- | --- |
| **For OsMDHAR4 over-expressing construct** | |
| *OsMDHAR4*-OE-F | ATGGGGCGGGCGTTCGTG |
| *OsMDHAR4*-OE-R | TCACCACCTACGGCGTTTCCTGC |
| **For subcellular localization construct** | |
| *OsMDHAR4*-YFP-F | GCTCTAGAATGGGGCGGGCGTTCGTG |
| *OsMDHAR4*-YFP-R | CGGGATCCCCACCTACGGCGTTTCCTGCC |
| **For qRT-PCR** |  |
| q*OsMDHAR4*-F | AGATTCGACAGGGAAGGTGA |
| q*OsMDHAR4*-R | CACGGATGCCAATACCAAC |
| q*Actin1*-F | TGGCATCTCTCAGCACATTCC |
| q*Actin1*-R | TGCACAATGGATGGGCCAGA |
| **For T-DNA insertion analysis** | |
| F1 | TCGTCGTCAATTCGTCATCT |
| R1 | TGTTTCCCTGCTGTGGTTTT |
| L4 | AATCCAGATCCCCCGAATTA |
| **For positive tests of transgenic plants** | |
| Hyg-F | GCTGTTATGCGGCCATTGTC |
| Hyg-R | GACGTCTGTCGAGAAGTTTC |
|  |  |
